# Diagnostic Accuracy of Ultrasound in Determining the Cause of Bilious Vomiting in Neonates

**DOI:** 10.5812/iranjradiol.8465

**Published:** 2012-11-20

**Authors:** Mehdi Alehossein, Siamak Abdi, Mohammad Pourgholami, Mohsen Naseri, Payman Salamati

**Affiliations:** 1 Advanced Diagnostic and Interventional Radiology Research Center (ADIR), Medical Imaging Center, Imam Khomeini Hospital, Tehran University of Medical Sciences, Tehran, Iran; 2 Department of Radiology, Bahrami Hospital, Tehran University of Medical Sciences, Tehran, Iran; 3 Department of Neurology, Shariati Hospital, Tehran University of Medical Sciences, Tehran, Iran; 4 Department of Pediatrics, Bahrami Hospital, Tehran University of Medical Sciences, Tehran, Iran

**Keywords:** Ultrasonography, Vomiting, Infant, Newborn, Bilious

## Abstract

**Background:**

Plain radiography and contrast radiologic studies are traditionally the main options in evaluating neonates presenting with bilious vomiting. While ultrasonography (US) is more available, its diagnostic accuracy is in question.

**Objectives:**

The purpose of this study is to determine the diagnostic accuracy of US in evaluating these patients with bilious vomiting.

**Patients and Methods:**

All neonates with bilious vomiting or bilious nasogastric tube drainage presented to a children’s hospital in a 1.5-year period were included. US were performed in all patients. The results were compared with clinical and radiological data and the final diagnosis. We used chi-square and Fisher’s exact tests for analysis.

**Results:**

The cause of bilious vomiting for 18 of the 23 included patients was surgical. All patients labeled as surgical candidates by US ended in surgery [positive predictive value (PPV) = 100%], while only 50% of the patients with inconclusive US were operated [negative predictive value (NPV) = 50%, Confidence Interval (CI) 95%: 29%-71%]. The sensitivity and specificity of US in diagnosing intestinal atresia (n = 9) was 89% [CI 95%: (68% - 100%)] and 100%. In cases with malrotation (n = 4) and midgut volvulus (n = 2), sonographic diagnosis was in concordance with final surgical diagnosis.

**Conclusion:**

This study suggested that in cases in which US makes a certain diagnosis, its accuracy eliminates the need for further diagnostic tests, but if it is inconclusive, further radiological contrast studies should be tried to make the final diagnosis.

## 1. Background

Bile vomiting in a neonate has traditionally been the cardinal sign of intestinal obstruction. In the absence of clinical signs or a plain abdominal film allowing a positive diagnosis, an upper gastrointestinal (UGI) contrast study is recommended to specifically exclude intestinal malrotation and volvulus (1, 2) which are the most emergent differential diagnoses. There are limitations for X-ray studies in neonates including radiation hazards, physical trauma such as the risk of hypothermia, difficult positioning and being time consuming (3). Meanwhile, there are studies which show that intestinal obstruction is not the cause of bilious vomiting in one half to two third of infants and most of these infants recover with no further sequel (4, 5). Performing routine UGI series for most of these neonates may be superfluous.

Several reports of the high accuracy of ultrasonography (US) in the diagnosis of midgut volvulus (6-8) made us interested to find whether using US as a safer and faster method may decrease standard UGI series usage in diagnosing the cause of bilious vomiting in neonates.

## 2. Objectives

The main aim of this study is to determine the diagnostic accuracy of US in evaluating patients with bilious vomiting.

## 3. Patients and Methods

### 3.1. Participants

Neonates with the history of bilious vomiting or bilious nasogastric drainage referred to Bahrami Children’s Hospital in Tehran were evaluated by ultrasonography. Neonates with definite diagnosis made by plain abdominal radiography were excluded. Neonates with a low position of nasogastric tube in the duodenum were not included in this prospective study.

### 3.2. Test Methods

**Figure 1:**
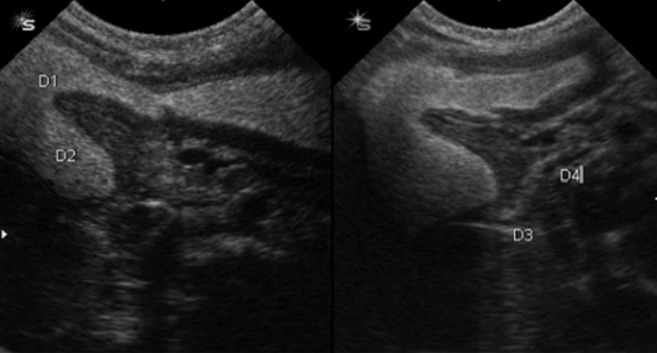
A 21-day neonate with bilious vomiting. Distended bulb (D1) and descending (D2) portions of the duodenum while horizontal (D3) and ascending (D4) portions of the duodenum are collapsed. Partial obstruction due to the web is nicely demonstrated.

**Figure 2:**
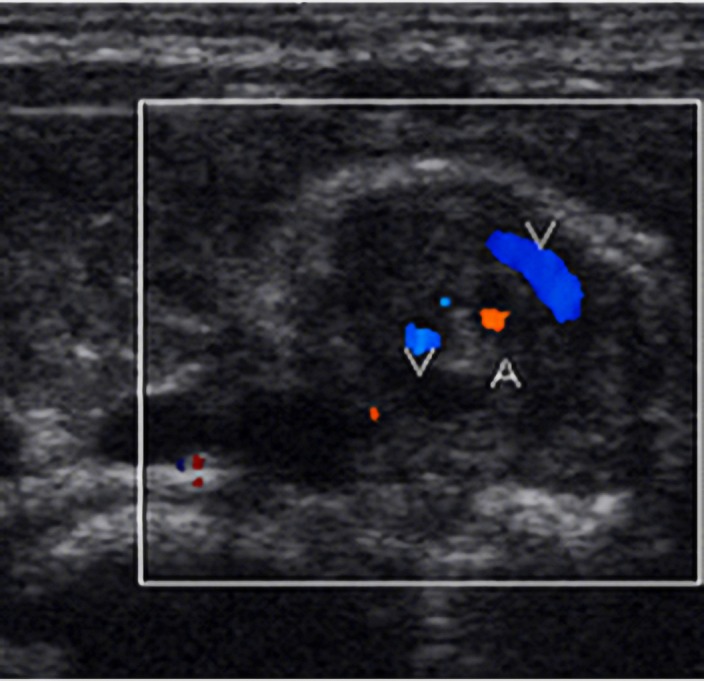
A 5-day male neonate with bilious vomiting Transverse color Doppler sonogram of the upper abdomen shows a whirlpool sign with a clockwise direction. Capital letter A indicates SMA and V indicate SMV.

Abdominal US were performed with a real time US unit (Siemens G50 with 7.5 convex and 10MHz linear transducers). Routine gray scale US was done by a pediatric radiologist. From the technical point of view, the baby was first located in the right lateral decubitus to displace gastric secretions toward the antropyloric portion and duodenum. In some stable cases, few milliliters of water were instilled via the nasogastric (NG) tube. We first characterized the antropyloric portion. The duodenal bulb was visualized as an arrowhead. Then we followed the descending portion of the duodenum lateral to the pancreatic head, the third horizontal portion (between the aorta and the superior mesenteric artery) and finally the fourth portion (cephalad and to the left). More distal bowel loops were followed by keeping the graded compression technique. Small and large bowels could be differentiated according to anatomic landmarks and their locations. The level of bowel obstruction may be determined by change in the caliber of the bowel loop or direct visualization of pathology (Figure 1). We reported details with clarification anatomy of the duodenum, distention or collapse of bowel loops, free fluid or other abnormalities. Color Doppler US was also conducted with attention to vascularization of the wall of the gastrointestinal tract, superior mesenteric vein/superior mesenteric artery (SMV/SMA) orientation and whirlpool sign (Figure 2).The whirlpool sign was recognized as a representative for midgut volvulus on transverse sonograms of the upper abdomen. The direction of the whirlpool was determined as clockwise or counterclockwise viewed from below the patient. When the transducer was moved in a craniocaudal direction on the abdomen, the whirlpool on the monitor whirled clockwise or counterclockwise according to the direction of the volvulus and the direction of the transducer motion. Surgical cases were implied in the sonographic report as well as inconclusive cases. Routine clinical care remained unchanged during the study period and some patients had UGI series too. A chart review was performed to document clinical information including the outcome that was obtained in the operating room or follow up in the neonatal ward.

### 3.3. Statistical Analysis

We used the SPSS software (version 16) for all data analyses. We analyzed data using Chi-Square and Fisher’s exact tests and statistical type one error was considered lower than 5%.

## 4. Results

### 4.1. Participants

The study was performed from December 1, 2007 to May 1, 2009. Twenty-three neonates (13 boys, 10 girls) considering inclusion criteria were identified. Their median age was 7.8 days (range, 1 to 27). Their median weight at birth and admission was 2922 and 2865 grams, respectively.

### 4.2. Test Results

Table 1 summarizes the findings of this study. Eighteen neonates (78%) had surgical pathology; jejunal atresia (n = 5), duodenal obstruction (n = 4), malrotation (n = 4), Hirschprung disease (n = 2) and miscellaneous (n = 4). One neonate had both malrotation and jejunal atresia according to the surgical report. Sensitivity and specificity of US in diagnosing intestinal atresia (n = 9) was 89% (CI 95%: 68% - 100%) and 100%. In cases with malrotation (n = 4) and midgut volvulus (n = 2), sonographic diagnosis was in concordance with the final surgical diagnosis. Miscellaneous cases consisted of esophageal atresia with tracheoesophageal fistula bypassing gastric vomitus (n = 1), hypertrophic pyloric stenosis with initial impression of bilious vomiting (n = 1), Hirschprung disease (n = 1) and adhesion band (n = 1). Five neonates (22%) were cured by medical management alone; three neonatal sepsis cases and two meconium plug disease cases. Seventeen neonates had plain abdominal radiography; normal (n = 5), gasless abdomen (n = 4), generalized intestinal distention (n = 3) and focal distention (n = 5).

**Table 1:** Summary of Characteristics, US and Final Diagnosis in 23 Neonates with Bilious Vomiting

Age, y	Gender	Weight	SMA-SMV Position	US Diagnosis	Management	Final Diagnosis
**6**	Male	2650	Normal	JA	Surgical	JA
**7**	Male	2350	AP	JA	Surgical	JA
**23**	Male	2150	AP	Malrotation	Surgical	Malrotation
**12**	Male	2000	Normal	NSF	Medical	Meconium plug
**11**	Male	2900	AP	NSF	Surgical	Adhesion bands
**3**	Male	4250	Normal	NSF	Medical	Meconium plug
**21**	Female	2450	Normal	Duodenal web	Surgical	Duodenal web
**3**	Male	2600	Normal	NSF	Surgical	HD
**2**	Female	2700	Normal	NSF	Surgical	EA
**8**	Female	2650	Reverse	MGV	Surgical	MGV
**4**	Male	3400	Normal	NSF	Surgical	ARM
**11**	Female	3350	Normal	Duodenal obstruction	Surgical	DA
**1**	Female	3150	Normal	JA	Surgical	JA
**2**	Female	2400	Normal	JA	Surgical	JA
**27**	Male	4400	Normal	HPS	Surgical	HPS
**1**	Male	2750	Normal	NSF	Surgical	HD
**2**	Female	2200	Normal	Duodenal obstruction	Surgical	Duodenal web
**11**	Female	2380	AP	NSF	Medical	Sepsis
**5**	Male	3550	Reverse	MGV	Surgical	MGV
**5**	Male	2250	AP	MGV	Surgical	JA and MGV
**3**	Male	3100	AP	Duodenal obstruction	Surgical	DA
**3**	Female	2780	Normal	NSF	Medical	Sepsis
**12**	Female	3900	Normal	NSF	Medical	Sepsis

Abbreviations: AP; anteroposterior, ARM; anorectal malformation, DA; duodenal Atresia, EA; esophageal atresia, HD; Hirschprung‘s disease, HPS; hypertrophic pyloric stenosis, JA; jejunal atresia, MGV; midgut volvulus, NSF; nonspecific findings, SMA; superior mesenteric Artery, SMV; superior mesenteric vein

Three patients had UGI series; two had malrotation and one was normal. In 12 cases (52.2%), there was concordance between sonographic diagnosis and final diagnosis. In 10 cases (43/5%), there was inconclusive sonographic diagnosis and there was one patient with more than one diagnosis at operation.

Superior mesenteric artery (SMA) - superior mesenteric vein (SMV) displacement was seen in all patients with malrotation. While the final diagnosis for both patients with SMA/SMV inversion was volvulus, it was the final diagnosis only for 33% of the patients with ventral SMV. All patients with volvulus had the whirlpool sign, although there was one false positive. All patients labeled as surgical candidates by US ended in surgery (PPV = 100%), while only 50% of the patients with inconclusive US were operated (NPV = 50%, CI 95%: 29% - 71%).

## 5. Discussion

Standard teaching in pediatric surgery mentions that bile vomiting in a neonate indicates intestinal obstruction until proven otherwise (1). There are studies which show that bilious vomiting in the neonatal period is not invariably associated with intestinal obstruction and the rate of intestinal obstruction may be as low as 38% (4, 5). Surgical intervention is necessary in 30% to 40% of neonates with bile vomiting (9). However, in our study, 78% of neonates with bilious vomiting required surgical intervention. As a referral pediatric surgical center, our hospital frequently admits patients with previous hospitalization which may account for the higher operation rate reported in the present study.

Although UGI series are considered as the modality of choice in the diagnosis of malrotation, there are shortcomings in the diagnosis or exclusion of malrotation with an up to 30% false positive rate, the risk of radiation exposure and the time consuming characteristic of the procedure. UGI series were performed only in three of our cases as routine clinical care and they remained unchanged during the study period, but color Doppler sonography was performed in all of them, referring to the policy of Schimanki et al. as the initial imaging study in children suspected of having midgut volvulus. Normally, the SMV is on the right side of the artery. In malrotation, the mesenteric vein is on the left side of the artery. However, this inversion is neither specific nor sensitive enough. A normal sonogram does not exclude malrotation (10). There are also other data which show that sonography is a good screening tool that effectively rules out malrotation at risk for volvulus (7). We had four cases (17.3%) of malrotation and three cases with the sonographic diagnosis of midgut volvulus of which finally two were surgically confirmed as midgut volvulus. The other case was as apparent over diagnosis of midgut volvulus by US that was detected as malrotation and jejunal atresia in surgery. This association was noted in literature as 28% of duodenal atresia and 19% of jejunoileal atresia had malrotation (2). The whirlpool sign in cases of volvulus refers to the sonographic appearance of the SMV, along with the bowel and mesentery wrapping in a clockwise mode around the SMA (11). Although it is accurate in the diagnosis of midgut volvulus, there are some case reports of the whirlpool sign being present in the absence of volvulus (12, 13). However, our case may be due to spontaneous devolvulation, considering that the simultaneous UGI series performed on this case was compatible with malrotation and volvulus (corkscrew sign). The counterclockwise whirlpool-like pattern of SMA and SMV was noted in another case with the final diagnosis of imperforated anus and no malrotation or volvulus of the midgut. There was one interesting case of esophageal atresia with tracheoesophageal fistula which bypassed the gastric contents to the proximal esophagus.

Another unusual case with hypertrophic pyloric stenosis was noted with initial impression of bilious vomiting. Contrast enema was performed when there was a high probability of lower obstruction in order to search for meconium ileus, ileal atresia, meconium plug syndrome, Hirschsprung’s disease and colonic atresia.

In conclusion, performing US in all neonates with bilious vomiting can depict surgical cases well if there are positive findings in the sonogram. In such cases, US can replace UGI series, resulting in decrease in the radiation dose, cost and time. The latter is very important in midgut volvulus which may be life threatening if not promptly operated.
